# Examining the Link between Air Quality (PM, SO_2_, NO_2_, PAHs) and Childhood Obesity: A Systematic Review

**DOI:** 10.3390/jcm13185605

**Published:** 2024-09-21

**Authors:** Barbara Siewert, Agata Kozajda, Marta Jaskulak, Katarzyna Zorena

**Affiliations:** 1 Environment and Health Scientific Circle, Medical University of Gdańsk, 80-210 Gdańsk, Poland; barbara.siewert@gumed.edu.pl (B.S.); a.kozajda@gumed.edu.pl (A.K.); 2 Department of Immunobiology and Environment Microbiology, Medical University of Gdańsk, 80-210 Gdańsk, Poland; katarzyna.zorena@gumed.edu.pl

**Keywords:** particulate matter (PM), PM_10_, PM_2.5_, PM_1_, NO_x_, SO_x_, PAHs, children, adolescents, obesity

## Abstract

**Background/Objectives:** Childhood obesity has emerged as a global health concern with profound implications for long-term health outcomes. In recent years, there has been increasing interest in the potential role of environmental factors in the development of childhood obesity. This comprehensive review aims to elucidate the intricate relationship between various components of air pollution and childhood obesity. **Methods:** We systematically analyze the existing literature from the past 5 years to explore the mechanistic pathways linking air pollution, including particulate matter (PM), nitrogen oxides (NO_x_), sulfur dioxide (SO_2_), and polycyclic aromatic hydrocarbons (PAHs), to childhood obesity. This systematic review examines 33 epidemiological studies on the link between air pollution and childhood obesity, published from 1 January 2018, to 31 January 2024. **Results:** Studies from counties with low overall air pollution noticed only low to no impact of the exposure to childhood obesity, unlike studies from countries with higher levels of pollution, suggesting that the mitigation of air pollutants can reduce the chance of it being a negative factor for the development of obesity. This relationship was noticed for PM_2.5_, PM_1_, PM_10_, NO_x_, and SO_2_ but not for PAHs, which showed a negative effect on children’s health across 10 out of 11 studies. **Conclusions:** This review underscores the need for interdisciplinary approaches to address both environmental and socio-economic determinants of childhood obesity. Efforts aimed at reducing air pollution levels and promoting healthy lifestyle behaviors are essential for safeguarding the health and well-being of children worldwide.

## 1. Introduction

In the 21st century, overweight and obesity in both adults and children are becoming a global epidemic. The World Health Organization (WHO) officially recognized obesity as “a chronic condition that requires treatment, promotes the development of other diseases and is associated with increased mortality” [1]. Obese individuals experience both short-term and long-term consequences. Even children, despite their young age, are at risk for dyslipidemia, hypertension, diabetes mellitus, non-alcoholic fatty liver disease, obstructive sleep apnea, psychosocial disturbances, an impaired quality of life, and a shorter life expectancy [2]. In the world population, obesity occurs in approximately 20% of children, adolescents, and adults. The prevalence of obesity in most developed societies has increased over the last 20 years. The latest results showed that childhood obesity is a major health problem, and the prevalence of overweight and obesity in school-age children in 2010 was 21–22% [3]. In the developmental age population, the data are as follows: approximately 155 million school-age children are overweight and obese; 30–45 million are children and adolescents aged 5 to 17, and 22 million are children under 5 years of age. There has been a significant increase in the incidence of obesity among preschool children [3]. However, among school-age children, excessive body weight was found to be more common in boys than in girls [4].

However, it is believed that, nowadays, genetic factors are responsible for the occurrence of obesity in only 25 to 45% [4,5], while changes in people’s lifestyle as well as global trends have a huge impact on the development of overweight and obesity. While there are two significant causes of childhood obesity, it would be an understatement to attribute the phenomenon solely to a sedentary lifestyle and an inappropriate diet. Overall, the greatest impact on the development of obesity has non-genetic factors—primarily environmental ones [6,7,8,9,10,11,12]. Urbanization, industrialization, and globalization contribute to the multifactorial character of civilization diseases. Urbanization heightens substantial health risks including air pollution and chemical, biological, and physical hazards that bring about illnesses. Simultaneously, it triggers changes in occupational activities and reinforces disproportions of socioeconomic status and social structures that can promote illnesses and inequalities in healthcare access [13].

According to statistics, the highest percentage of obese people occurs among those with a lower socio-economic status [1]. Among such people, the awareness of the problem of obesity, its causes, and its consequences is often lower. Rather intuitively, overweight and obesity were reported to occur in economically developed countries due to a greater supply of food and reduced physical activity [13]. However, the disparities between countries resulting from recent urbanization or urban sprawl dictate the obesity growth rates. They have flattened or started decreasing in the majority of High-Income Countries, whereas in Low- and Middle-Income Countries, drastic increases have been seen in recent years [13].

According to the WHO, environmental risks cause 12% of the global burden of disease, with air pollution ranking first [14,15,16]. It exists as a burning problem leading to numerous health problems in low- and middle-income countries, where 92% of air pollution-related deaths occur. Air pollution may be regarded as an obesogenic factor and constitutes a common threat worldwide [16]. Most important air pollutants include particulate matter (PM_1_, PM_2.5_, PM_10_, etc.), carbon monoxide (CO), nitrogen oxides (NO_x_), sulfides, and Polycyclic Aromatic Hydrocarbons (PAHs) [17].

There is growing evidence showing that air pollution may be associated with obesity development [2]. Several mechanisms for this have been postulated, including changes in the basal metabolism, inducing systemic inflammation, oxidative stress, and hormone disruption [1,2,18], increases in brain inflammation, including microglial activation and anxiety, leading to increased caloric intake [3], and influences on child behavior, such as physical activity levels and eating habits [2]. In humans, outdoor and indoor air pollution is the root of morbidity, influencing respiratory diseases as well as inducing inflammation, cardiovascular diseases, obesity, diabetes, cancer, Alzheimer’s Disease, and premature mortality [19]. Moreover, once obesity, hypertension, or diabetes is developed in an individual, they become more susceptible to air pollution exposure, showing more drastic increases in markers of inflammation (Table 1) [20].

Since children have relatively weaker lung defenses and immunity, they are even more susceptible to the negative effects of air pollution than adult individuals [24,25], notwithstanding the fact that they may be exposed to higher levels of air pollutants due to their increased outdoor time and higher levels of physical activity out there [26]. In such polluted environments, the beneficial effects of outdoor physical activity are diminished and have no impact on cardiopulmonary fitness or any health benefits whatsoever [27,28]. Moreover, they lack control over their diet, surroundings, and living conditions and may have little understanding of the consequences of developing obesity [4].

In this way, they reflect faulted social, economic, education, and urban planning and agricultural policies; thus, this childhood obesity should be taken seriously into account and managed more efficiently to provide early prevention [28].

Despite some developments in preventative measures encompassing technological solutions and spreading environmental awareness in many countries across the world, air pollution poses a tremendous threat to mankind [15]. Our previous research showed that high exposure to gaseous pollutants and particulate matter in ambient air may be one of the factors contributing to the risk of developing diabetes mellitus type 1 (T1DM) in children [29,30]. Currently, our systematic review provides a valuable summary of the newest knowledge about the association between air pollution and childhood obesity. The primary aim of this review article is to comprehensively investigate and analyze the association between various components of air pollution, including particulate matter (PM), sulfur dioxide (SO_2_), nitrogen dioxide (NO_2_), and polycyclic aromatic hydrocarbons (PAHs), and childhood obesity. Understanding this relationship is of paramount importance due to the rising prevalence of childhood obesity worldwide.

## 2. Methods

The systematic review takes into consideration a wide variety of studies that explore the connection between air pollution and childhood obesity. The focus was on the articles published between 1 January 2018 and 31 January 2024 (a 5-year overview of findings).

Criteria for Defining Childhood Obesity: In the included studies, childhood obesity was defined using various criteria. Most studies utilized Body Mass Index (BMI) percentiles specific to age and sex, as recommended by the Centers for Disease Control and Prevention (CDC) and the World Health Organization (WHO). Specifically, children with a BMI at or above the 95th percentile for their age and sex were classified as obese [31,32]. 

Search Strategy: the literature search was conducted using the PubMed, Scopus, and WebOfScience databases. The search strategy employed specific keywords related to air pollutants and childhood obesity, including both full terms and abbreviations. The search terms included “PM” or “particulate matter” (1, 2.5, and 10 μm), “NO_x_” or “nitrogen oxides”, “SOx” or “sulfate oxides”, and “PAH” or “polycyclic aromatic hydrocarbons” combined with “children obesity”, “child obesity”, or “adolescent(s) obesity”.

Inclusion Criteria:

- Language: Only studies published in English were considered.
- Publication Date: Studies published between 1 January 2018, and 31 January 2024.
- Population: Participants under 18 years of age.

Study Design: Original research studies including clinical, longitudinal, and cross-sectional studies that provided real-life observational data were prioritized. High-quality review articles, such as systematic reviews and meta-analyses, were only included in the introduction to offer a broader context or explain the possible mechanisms of action, provided they met the same inclusion criteria.

Focus: The studies had to explicitly investigate the relationship between air pollution and childhood obesity.

Exclusion Criteria: Studies in which obesity was considered a secondary outcome without clear or correlated measurement parameters related to air pollution; non-peer-reviewed articles, editorials, and opinion pieces; and studies relying solely on computer models or animal experiments.

**Figure 1:**
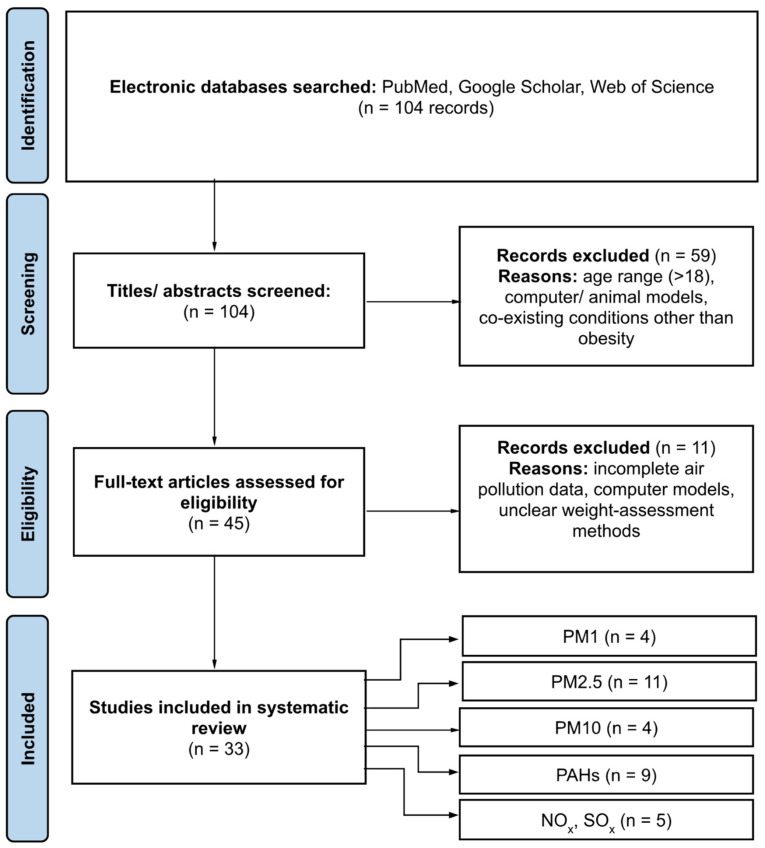
PRISMA flowchart [34].

Study Selection and Data Extraction: The study selection process was carried out by two independent reviewers. Discrepancies were resolved through discussion or, when necessary, consultation with a third reviewer. The PRISMA flow diagram (Figure 1) illustrates the study selection process. Data extraction was performed using a standardized form, capturing study characteristics, population details, exposure metrics, outcome measures, and key findings. Quality assessment of the included studies was conducted using established criteria, ensuring the reliability of the evidence.

Data Synthesis: The studies were then categorized by the type of air pollutant and analyzed both chronologically and by the strength of their reported effects. The data were synthesized to identify patterns and draw conclusions regarding the relationship between various air pollutants and childhood obesity.

Quality Assessment: Each study included in the review underwent a quality assessment to evaluate the risk of bias. This assessment was based on predefined criteria tailored to the study designs included, such as the sample size, methodology robustness, and accuracy of exposure and outcome measurements. To assess the quality of the included studies, we employed the Newcastle–Ottawa Scale (NOS) for non-randomized studies, which evaluates studies based on three broad criteria: the selection of study groups, the comparability of groups, and the ascertainment of either the exposure or outcome of interest [33]. The NOS assigns a maximum of nine stars, with higher scores indicating higher quality. Studies scoring seven or more stars were considered of high quality, while those with fewer stars were evaluated for potential bias. These results are discussed in the context of the overall findings in the tables and the Section 4.

## 3. Associations between the Exposure to Air Pollution and Childhood Obesity

### 3.1. PM_2.5_

**Figure 2:**
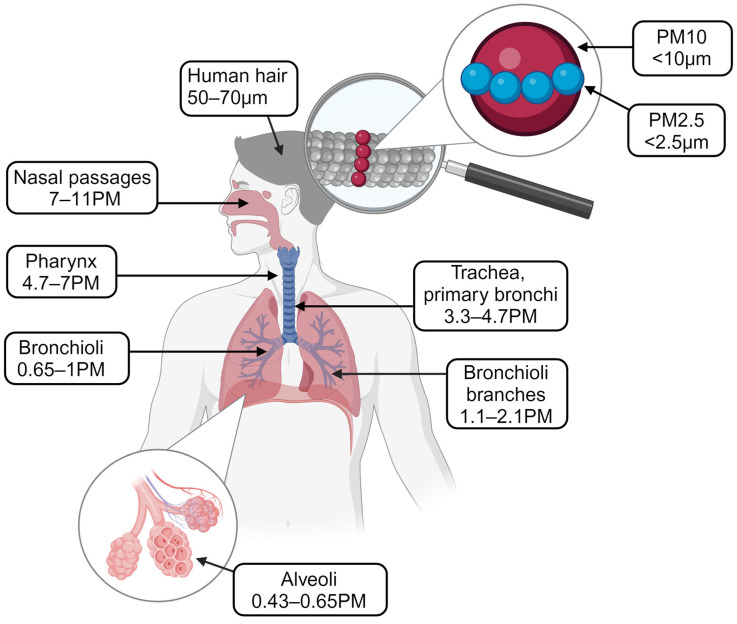
Comparison of PMs’ perimeters and areas where PMs penetrate the respiratory system [36]. PM_10_ is known to penetrate the nasopharynx and start inflammatory reactions in the upper levels of the respiratory tract. PM_2.5_ appears to have a particularly adverse effect due to its size and ability to travel deep within the respiratory system. PM_1_ is thought to penetrate the lowest portions of the respiratory system (such as the bronchioli and alveoli), although many details of its nature and mechanisms are still to be explored. [Created with BioRender.com accessed on 6 April 2024. This figure is licensed under BioRender’s Academic License Terms and is intended for use in this publication only. Agreement number: MQ278AXY29].

The distinct properties of PM_2.5_ make it particularly harmful when compared to larger particles such as PM_10_. Due to its small size, PM_2.5_ can penetrate deep into the lungs and enter the bloodstream, allowing it to travel to various organs [35]. This characteristic enables PM_2.5_ to have a more widespread and profound impact on the body, contributing to systemic inflammation, oxidative stress, and endocrine disruption. These mechanisms are increasingly being linked to metabolic changes and the development of obesity, particularly in children, who are more vulnerable to environmental pollutants. Figure 2 shows potential sites of absorbance of air contaminants into the system [36].

The significant attention PM_2.5_ has received from researchers is driven by its global prevalence in the environment and its demonstrated adverse health effects [17]. Numerous epidemiological studies have linked PM_2.5_ exposure to a range of health outcomes, including respiratory and cardiovascular diseases, as well as metabolic disorders like obesity [2,20]. The emerging evidence connecting PM_2.5_ to childhood obesity has sparked particular concern due to the long-term health implications for affected children, such as an increased risk of developing chronic diseases later in life [25].

The main epidemiological studies that explored the potential association of PM_2.5_ exposure and childhood obesity in the past 5 years are presented in Table 2 [37,38,39,40,41,42,43,44,45,46].

A cohort study conducted by Tong et al. [47] has demonstrated a dose–response relationship between the PM_2.5_ level and obesity in children aged 6 to 8 years, with a particularly strong link to central obesity. Similarly, exposure to PM_2.5_ among school-age children was associated with an increased BMI Z-score, waist circumference and waist-to-height ratio, and higher prevalence of not only central but also general obesity. The results were more pronounced in boys than in girls, except for general obesity. Interestingly, reductions in PM_2.5_ levels after the launch of environmental protection measures showed that the risk of overweight/obesity decreased by 8% with a 1 μg/m^3^ increment in PM_2.5_, but the likelihood of obesity was smaller in females than in males (Liang et al., 2022) [48]. Two studies in Mexico discovered links between exposure to PM_2.5_ of similar levels and obesity as well as glucose dysregulation [34,47]. The average concentration of 22.4 µg/m^3^ PM_2.5_ has led to changes in the levels of HbA1c. Each 10 µg/m^3^ increase in PM_2.5_ was likely to double the probability of developing obesity in adolescents. A large-scale study of Peruvian children aged 0.5 to 5 years has suggested that intrauterine and extrauterine exposures of an increment of 10 μg/m^3^ significantly increase the odds of overweightness or obesity using the WHO reference value for the sex and height scheme [48]. However, Fioravanti et al. (2008) found no relationship between exposure to PM_2.5_ of a time-averaged concentration of 19.5 µg/m^3^ assessed by geocoding and overweightness or obesity in a group of 500 children at 4-year and 8-year follow-ups measuring the waist circumference, waist-to-hip ratio, and total and HDL cholesterol [49].

In contrast, a study focusing on the Boston area, with a much lower postnatal exposure to PM_2.5_ (the concentration being only 8.5 µg/m^3^, on average) has modified the weight trajectory in the first 5 years of life: there was stronger association between weight and age in males [35]. Moreover, as a result of an investigation of 3460 participants, a positive association between the exposure and weight was observed in the first 3 years of life, becoming negative afterwards. Interestingly, low-birth-weight infants were found to be more vulnerable to normal-weight babies [50]. In another study, similar conclusions have been drawn concerning preterm babies [51]. A positive correlation between prenatal airborne pollution exposure and weight for length and body mass indices was found among 1-year-old children. In light of Zhang’s Chinese cross-national study, average levels of PM_2.5_ pollution ranging from 46.9 to 81.0 µg/m^3^ show no evidence for an association between airborne pollution and hypertension-elevated HDL-C or TG levels; nevertheless, they impacted the odds of developing metabolic syndrome as well as abdominal obesity, which were even higher than those in the former study [38].

**Table 2:** Associations between PM_2.5_ exposure and childhood obesity.

Author, Year	Location	Age [Years]	Cohort Size	Biomarkers	Main Findings
Fioravanti, 2018 [49]	Italy	0 follow up at 4 and 8	500	waist circumference, waist-to-hip ratio, total and HDL cholesterol	No evidence of an association was found between exposure to air pollutants and overweight/obesity in children.
Moody, 2019 [52]	Mexico	0–7	365	HbA1c levels	Prenatal and perinatal exposures to PM_2.5_ are associated with changes in HbA1c, which are indicative of glucose dysregulation, in early childhood.
Tamayo-Ortiz, 2021 [35]	Mexico	-	2219	weight and height were measured, following the cut-off points in the WHO guidelines, and for children and adolescents, age-specific guidelines from the Mexican Social Security Institute that follow age-specific WHO guidelines were used.	An almost twofold increase in the odds of obesity for each 10 µg/m^3^ of PM_2.5_. The association was strongest for adolescents.
Zhang, 2021 [53]	China	10–18	9897	glucose oxidase, TG, FBG, HDL-C, abdominal obesity	No positive associations between air pollutants and hypertension, HDL-C, or TG; MetS subjects were more likely to be boys, to have a higher BMI, and to have a family history of type 2 diabetes, hypertension, obesity, or cerebrovascular disease when compared to participants without MetS. The odds ratio of MetS is associated with a 10 μg/m^3^ increase in the two-year average of 1.31 for abdominal obesity.
Zhou, 2021 [51]	China	0–1	10,547	weight and length, weight for length (WFL), and body mass index (BMI), with overweight and obesity (OWOB) defined based on WHO Standards	Positive associations of prenatal PM_1_ and PM_2.5_ exposure with the WFL and BMI Z-score for one-year-old children, which could be partly explained by preterm birth. 10 μg/m^3^ increase in prenatal exposure to PM_2.5_. It was significantly associated with an increase in the WFL Z-score for one-year-old children. Similar associations were found for the BMI Z-score.
Zhang, 2021 [38]	China	7–18	44,718	BMI Z-score, waist circumference, waist-to-height ratio	Exposure to PM_2.5_ was associated with an increased BMI Z-score, waist circumference, and WHtR and a higher prevalence of both general and central obesity. Stronger associations were observed for particles, especially PM_1_ and PM_2.5_, than for gaseous pollutants, e.g., NO_2_. The results were more pronounced in boys than in girls, except for general obesity.
Wu, 2022 [39]	China (SNEC)	6–18	47,990	child age- and sex-specific z-scores for body mass index (BMI Z-score) and weight status were generated using the WHO growth reference	Exposure was associated with a greater childhood BMI Z-score and a higher likelihood of obesity. Similar associations were observed for PM_1_, PM_2.5_, and PM_10_ and were greater in boys and children living close to roadways.
Paz-Aparicio, 2022 [37]	Peru	0.5–5	65,232	values for height and weight for each sex assessed using the WHO guideline	A significant association between O/O and extrauterine and intrauterine PM_2.5_ exposure for an increment of 10 μg/m^3^.
Vanoli, 2022 [50]	USA	0–5	3460	weight	PM_2.5_ significantly modified the association between age and weight in males, with a positive association in children younger than 3 years and a negative association afterwards. Low-birthweight babies are more susceptible than normal-weight infants
Liang, 2022 [48]	China	7	2105	BMI: the standards from the growth charts of the China Centers for Disease Control (CDC) were then used to calculate the BMI z score (BMIz); overweight and obesity were diagnosed based on sex-specific BMI-for-age growth charts from the China CDC.	The results showed that the risk of overweight/obesity decreased with a 1 μg/m^3^ decrease in PM_2.5_ after adjusting for covariates. The reduction in the risk of obesity was larger in females than in males
Tong, 2022 [47]	China	6–8	4284	BMI	Higher levels of accumulating exposure to PM_2.5_ were associated with an increased childhood obesity index, and the effect was the most significant for WHtR compared to BMI and BMIz. This effect was more pronounced in boys than in girls, except for WHtR, and it was the most significant under the PM_2.5_ exposure period from pregnancy to 6 years old. Dose–response relationship between PM_2.5_ exposure and childhood obesity, especially central obesity

### 3.2. PM_1_

The main studies that explored the potential relationship between the exposure to PM_1_ and childhood obesity within the past 5 years are presented in Table 3 [37,50,51,52]. An exposure to PM_1_ of an average concentration of 50.8 µg/m^3^ induced short-term effects among participants aged 9–18: the associations between PM_1_ and systolic blood pressure and diastolic blood pressure were more pronounced in females, younger individuals, participants already overweight or obese, and those with insufficient physical activity (Wu et al., 2020) [39]. Accordingly, in a Wu et al. study from 2022, increments of 10 mg/m^3^ resulted in an increase in the systolic pressure of 2.56 mmHg and, in turn, increased the probability of hypertension by 61% [40]. Zhang et al. found out that PM_1_ is a leading agent among air pollutants (at a concentration of 46.9 µg/m^3^) in causing an increased BMI Z-score, waist circumference and weight-to-height ratio, along with a higher prevalence of both general and central obesity [53]. Zhang et al. found no evidence of a correlation between developing metabolic syndrome and PM_1_ levels; however, a 10 µg/m^3^ increase in PM_1_ was linked with elevated FBG measures among the 10–18 age group of 9897 participants (Zhang et al., 2021) [38]. A 10 μg/m^3^ increase in prenatal exposure to PM_1_ was significantly associated with a 0.105 increase in the WFL Z-score for one-year-old children. In another Chinese study, the positive associations of prenatal PM_1_ exposure with WFL and the BMI Z-score for one-year-old children, although moderated by preterm birth, were not negligible (Zhou et al., 2021) [53] (Table 3).

### 3.3. PM_10_

The main studies that explored the potential relationship between the exposure to PM_10_ and childhood obesity within the past 5 years are presented in Table 4 [43,44,51,54].

de Bont and his co-workers, in 2019, examined over 2500 children aged 7–10 years in Barcelona, Spain [41]. More than three-quarters of the participants were exposed to PM_10_ levels higher than the WHO official recommendation, being <20 μg/m^3^. According to the acquired z-BMI scores, a 5.6 μg/m^3^ increase in PM_10_ at-home levels was associated with a 10% increase in the odds of being overweight. A study conducted by the same author in 2021 confirmed the previous findings. Having examined over 400,000 children aged 2 to 5, it was thought that the increased exposure to PM_10_ is connected with a 2–3% risk of obesity development (which seems to be much more detailed of a finding than the previous study). A similar percentage of participants experienced the PM_10_ pollution level exceeding WHO guidelines. Interestingly, the correlation between air pollutants and obesity was visibly stronger for children from more deprived areas compared to those of the middle class. A study performed by Zhang et al. in 2021 on almost 45,000 Chinese children under 18 shows similar results [38]. Not only the BMI-score but also the waist-to-height ratio is considered to be influenced by PM_10_. The increase in the BMI z-score was 0.11 per 10 μg/m^3^ increment in annual average concentrations of PM_10_. The association seemed to be more visible in the boys’ groups than in girls, except for general obesity (even distribution of results). However, particles of a smaller diameter such as PM_2.5_ and PM_1_ were found to have a greater effect than PM_10_, possibly due to their ability to penetrate deeper parts of the pulmonary system. Huang et al. (2022) provides results largely consistent with previous findings [42]. The study additionally considered the skinfold thickness and body fat percentage in order to classify participants as overweight or obese. It is suggested that PM_10_ with 10 μg/m^3^ increment has the potential to elevate the risk of obesity globally (Lin et al., 2022) [43]. Although other authors indicate that the significant changes occur when the concentration exceeds 20 μg/m^3^, Zheng et al. (2024) presents the outlook that the significant rise in the risk of obesity takes place at the point of 50 μg/m^3^, considerably higher than what was established by previous authors [44]. It is indicated that PM_10_ exposure increases the probability of being overweight or obese, especially in Asia, which can be caused by a cultural-based difference in the definition of obesity.

Parasin et al. (2021) found an increase in the risk of developing obesity in children caused by PMs exposure, and the effects of PM_10_ after the follow-up period of 4 years were found to be statistically insignificant [45]. The author points to the wide variety of mechanisms involved in PM_2.5_ and PM_1_ exposures compared to PM_10_. Similarly, no association between exposure to pollutants and obesity was found in an Italian birth cohort [49]. The study used various obesity-related parameters like BMI, waist circumference, waist-to-hip ratio, and total and HDL cholesterol, and none were proven to consistently change with PM_10_ exposure. The conclusion is that the knowledge of the obesogenic qualities of air pollution remains quite limited, and further studies need to be conducted.

### 3.4. NO_x_ + SO_2_

The main epidemiological studies that explored the potential link between NO_x_ and SO_2_ exposure and childhood obesity are presented in Table 5 [38,49,53,55,56]. In light of China’s national cross-sectional study, each increase of 10 µg/m^3^ of NO_2_ was associated with a heightened prevalence of FBG among the 10–18 age group [38]. Moreover, in another study by Zhang et al., exposure to NO_2_ was associated with an increased BMI Z-score, waist circumference, and weight-to-height ratio and a greater share of both general and central obesity; yet, NO_2_, being a gaseous pollutant, showed a weaker relationship than that of particulate matter. However, a study by Fioravanti et al. showed no correlation between air pollutant levels and overweight or obesity in children in an Italian birth cohort study with the lowest cohort size among the analyzed articles despite a seemingly high concentration of 69.8 µg/m^3^ [53]. Similar results were shared in a study examining exposure to traffic-related air pollution during fetal life and child obesity in 4-year-olds in Sweden. There was no correlation between NO_x_ concentration and overweightness or obesity at the follow-up; moreover, an interesting finding was weak evidence of a link between NO_x_ levels and being underweight [55]. This might be attributed to the low and medium pollution levels in this country and the study including only one constituent of traffic-related air pollution. A study investigating long-term exposure to NO_2_ and SO_2_ among children in Hong Kong includes four follow-ups at 9, 11, 13, and 15 years. The trajectory of weight is different, with a higher BMI at 9,13 and 15 years due to heightened exposure to NO_2_, whereas lower BMI values at 13 and 15 years were linked to low SO_2_ exposure [56].

### 3.5. PAHs

The main epidemiological studies exploring the association between childhood obesity and the exposure to PAHs are presented in Table 6 [57,58,59,60,61,62,63,64,65]. A study investigating the consequences of the exposure of 186 participants aged 6–18 to polycyclic aromatic hydrocarbons in Iran has reached a conclusion that most of the evaluated PAHs enhanced the risk of cardiometabolic risk factors and excess weight. Specifically, exposure to the compounds 2-naphtol, 9-phenanthrol, and ∑ OH-PAH was associated with an increased risk of obesity for participants without cardiometabolic risk factors [57,58].

The urinary PAHs concentration and a higher WHtR, an indicator of central obesity even at 3–5 years, were linked in a Canadian study with a sample size of 3667. Surprisingly, in the same age group, the BMI and the matrix were not associated. Overall, among the total study population between 3 and 18 years of age, the participants placed in the highest quadrille for total PAH metabolites or naphthalene only had a three-times-higher risk of developing central obesity than those in the lowest quartile [60,61]. An American study from 2019 supports the previous findings and suggests a positive association of air PAH contamination with childhood BMI and BMI Z-scores at the ages of 5 to 10. Moreover, the growth trajectory of study participants was observed to increase across follow-up visits until age 11 [59].

Higher ambient PAH contamination influenced the levels of HbA1c, systolic blood pressure, and oxidative stress in the US, with a cohort size of 299, who were aged 6–8 years. It was found that both short- and long-term outdoor residential exposures to traffic-related air pollution including PAHs are associated with biomarkers indicative of a risk for metabolic syndrome [63].

Another study investigated the relationship between indoor air pollution exposures and obese anthropometric indices among Chinese schoolchildren. The study suggests that exposure to more than or equal to three types of indoor air pollution including PAHs increases z-BMI and the overall risk of overweight or obesity. Moreover, the authors discovered a dose–response relationship between the exposure and the aforementioned indices [63].

The only study that has not found an association between PAHs and higher childhood adiposity had the smallest sample size of 198 and monitored relatively low levels of elemental carbon attributable to traffic only during the prenatal period. The follow-up was performed after 7–8 years [61]. Notwithstanding the foregoing, its differing results may have been largely dictated by severe limitations.

## 4. Discussion

Obesity, being a complex and multifactorial illness defined as “abnormal or excessive fat accumulation that presents a risk to health” by WHO, poses a threat to people at different ages. With a dramatic increase in obesity rates among the general population, the problem of the prevalence of childhood obesity is likely to be an underlying cause for a major incoming health downfall including premature death and disability [65]. Current research has shown that obese children experience both short-term and long-term consequences, e.g., cardiovascular diseases, diabetes, and certain types of cancers, with an earlier onset or greater probability than the general population [48,60,66].

Numerous factors are likely to put children at risk for developing obesity; each and every one of them is dangerous on its own, but researchers suggest that they often come together. An unhealthy diet and a habit of fast food nutrition in particular as well as less physical activity are the main causes of an increased prevalence of obesity, particularly among adolescents living in cities [65,67].

There are socioeconomic factors, including a low socioeconomic status, low parental education, non-parental caregivers, a lower fruit-eating frequency, short sleeping hours, and parental obesity. Maternal-Related Factors even before the childbirth especially seem to play an important role in the process: research has shown that a higher pre-pregnancy BMI directly correlates with childhood obesity [68].

Psychological aspects remain particularly significant causes of obesity: adverse childhood experiences are social determinants of health [66]. Comorbidities are reported to accumulate: unhealthy weight was continuously present among children with comorbidities such as autism spectrum disorders and individuals with sleep and affective problems [69].

Moreover, sleep duration and quality are deemed significant risk factors for childhood obesity. A meta-analysis conducted by Han et al. reported that an increased risk of childhood obesity is accompanied by short sleeping durations [70].

### 4.1. Mechanisms by Which Air Pollution Affects Obesity

The potential mechanisms linking air pollution exposure to childhood obesity involve complex interplays between environmental factors, physiological responses, and behavioral patterns. The 33 reviewed manuscripts in Table 1–5 show several mechanisms by which air pollution has an impact on obesity. First, airborne pollutants such as PM, SO_2_, NO_x_, and PAH can induce systemic inflammation and oxidative stress upon inhalation. These inflammatory responses can disrupt metabolic homeostasis, leading to insulin resistance, dyslipidemia, and altered adipokine secretion, all of which contribute to adiposity and weight gain in children [38]. Additionally, exposure to air pollution constituents has been associated with alterations in the gut microbiota composition, which can influence energy metabolism and adipose tissue development [70]. Furthermore, air pollution exposure may also impact neuroendocrine pathways regulating appetite and satiety [38]. The inhalation of pollutants can affect central nervous system function, including regions involved in appetite regulation such as the hypothalamus. The disruption of these regulatory pathways may lead to dysregulated eating behaviors, increased food intake, and, ultimately, weight gain. Moreover, chronic exposure to air pollution has been linked to disruptions in sleep patterns, which can further exacerbate metabolic dysfunction and weight gain in children [71,72].

Beyond physiological mechanisms, our review shows that environmental and socio-economic factors play significant roles in the relationship between air pollution and childhood obesity [73]. Children living in areas with high levels of air pollution often experience limited access to outdoor play spaces and opportunities for physical activity, leading to sedentary lifestyles and reduced energy expenditure. Moreover, socio-economic disparities exacerbate these effects, as low-income communities are disproportionately exposed to higher levels of air pollution and face barriers to accessing healthy food options and healthcare resources [13]. Overall, the studies referred to in Table 1, Table 2, Table 3, Table 4 and Table 5, performed on children living in countries with relatively low pollution levels, showed no association or a weak association between the air pollution and the risk of obesity in children, whereas in developing countries with higher pollution levels, this relationship was prominent.

Exposure to fine particulate matter (PM_2.5_) has been implicated in the development of childhood obesity through several mechanisms. PM_2.5_ inhalation was demonstrated to induce systemic inflammation and oxidative stress. These inflammatory responses trigger the release of pro-inflammatory cytokines and adipokines, disrupting metabolic homeostasis. Chronic inflammation is associated with insulin resistance, dyslipidemia, and impaired glucose metabolism, all of which contribute to adiposity and weight gain in children [74]. Moreover, PM_2.5_ exposure has been linked to alterations in adipose tissue biology. The reviewed research in our study has shown that exposure to PM_2.5_ can lead to adipocyte hypertrophy and hyperplasia, promoting the accumulation of fat mass. Additionally, PM_2.5_-induced inflammation can disrupt adipose tissue function, impairing the secretion of adipokines involved in energy metabolism regulation [75]. Obese children with asthma are more vulnerable to air pollution, especially fine particulate matter [49]. Children with an abnormal body weight and asthma breathe at higher tidal volumes that may increase the efficiency of PM_2.5_ deposition in the lung. This finding may partially explain why obese children with asthma exhibit greater sensitivity to air pollution. PM_2.5_ can also affect gene expression in mitochondria in brown adipose tissue, resulting in an increased production of reactive oxygen species in brown fat stores, which lead to metabolic dysfunction and susceptibility to lipid metabolism and glucose metabolism [49]. Second, the inflammatory response that is triggered by air pollutants can lead to vascular damage as well as insulin resistance and can also have an impact on body weight [47]. Studies also found that the occurrence of sleep-disordered breathing (SDB) was related to exposure to air pollutants [34,39]. Those who lived in regions with high NO_2_ and PM_2.5_ levels were much more likely to suffer from SDB, which in turn caused mental and physical health disparities including increased weight [50].

Frequent contact with coarse particulate matter (PM_10_) has also been implicated in the development of childhood obesity through various mechanisms, although the effects may differ slightly from those of PM_2.5_. In addition to its effects on adipose tissue, PM_10_ inhalation can also impact respiratory function in children. Respiratory distress caused by PM_10_ exposure may lead to decreased physical activity levels and impaired exercise tolerance, contributing to a sedentary lifestyle and weight gain. Additionally, PM_10_ exposure has been associated with respiratory infections and asthma exacerbations in children, which can lead to decreased lung function and physical activity levels, both of which are risk factors for obesity (Table 1 and Table 2).

The presence of ultrafine particulate matter (PM_1_) presents unique challenges and potential mechanisms in relation to childhood obesity. PM_1_ particles, being smaller in size than PM_2.5_ and PM_10_, have a greater surface area per unit mass, allowing them to penetrate deeper into the respiratory system and potentially enter the bloodstream directly. This characteristic enhances their ability to induce systemic inflammation and oxidative stress, which are key drivers of metabolic dysfunction and adiposity in children [51]. One significant mechanism by which PM_1_ may influence childhood obesity is through its impact on adipose tissue biology. Ultrafine particles can infiltrate adipose tissue more readily compared to larger particles, leading to localized inflammation and dysfunction within adipocytes. This disruption can impair adipokine secretion and adipose tissue remodeling, contributing to adiposity and weight gain [50]. Moreover, PM_1_ particles have been shown to cross the blood–brain barrier and affect central nervous system function. Neuroinflammation and oxidative stress induced by PM_1_ exposure may disrupt hypothalamic pathways involved in appetite regulation, leading to dysregulated eating behaviors and increased food intake, ultimately promoting obesity in children [52]. Furthermore, PM_1_ inhalation has been associated with cardiovascular effects, including endothelial dysfunction and impaired vascular reactivity. These cardiovascular effects may exacerbate metabolic dysfunction and adiposity, further contributing to the development of obesity in children. Additionally, similar to other particulate matter fractions, PM_1_ exposure can exacerbate respiratory conditions such as asthma, leading to decreased lung function and physical activity levels, which are risk factors for obesity [37].

Overall, immature metabolic pathways can increase susceptibility to environmental damage. The mechanisms linking various types of particulate matter (PM) exposure to hypertension are not yet fully understood. It is hypothesized that in the short term, PM exposure disrupts the autonomic nervous system and causes direct local oxidative and inflammatory effects on blood vessels. Over the long term, these effects may combine with chronic, secondary systemic inflammation and oxidative stress, potentially leading to elevated blood pressure. The diameter of PM plays a crucial role in its health impacts, as finer particles can enter the bloodstream directly, triggering a secondary proinflammatory response [35,37,47,48,54].

For NO_x_, one significant mechanism by which NO_x_ may influence childhood obesity is through its role in oxidative stress and inflammation. NO_x_ exposure can induce the production of reactive oxygen species (ROS) and reactive nitrogen species (RNS) in the body, leading to oxidative damage to cells and tissues. This oxidative stress triggers inflammatory responses, characterized by the release of pro-inflammatory cytokines and chemokines, which can disrupt metabolic homeostasis and promote adiposity. NO_x_ exposure has also been associated with endothelial dysfunction and impaired vascular health, particularly in children with pre-existing cardiovascular risk factors. Endothelial dysfunction can lead to reduced NO_x_ bioavailability and impaired vasodilation, contributing to hypertension and metabolic disturbances associated with obesity [51]. Additionally, NO_x_ exposure may influence adipose tissue biology and energy metabolism. Animal studies have suggested that NO_x_ exposure can alter adipocyte differentiation and function, leading to adipocyte hypertrophy and adipose tissue inflammation. Furthermore, NO_x_-induced inflammation may impair insulin signaling pathways in adipose tissue, exacerbating insulin resistance and metabolic dysfunction. NO_x_ exposure has also been linked to alterations in gut microbiota composition, which can influence energy harvest from the diet and adiposity. Dysbiosis induced by NO_x_ exposure may promote inflammation and metabolic disturbances, contributing to obesity development in children [54].

One significant mechanism by which SO_2_ may influence childhood obesity is through its pro-inflammatory effects. SO_2_ exposure can induce oxidative stress and inflammation in the respiratory system and other tissues. This inflammatory response triggers the release of pro-inflammatory cytokines and chemokines, which can disrupt metabolic homeostasis and contribute to adiposity [41]. Moreover, SO_2_ exposure has been linked to respiratory conditions such as asthma exacerbations and airway inflammation in children. Respiratory distress caused by SO_2_ exposure may lead to decreased physical activity levels and exercise intolerance, which are risk factors for obesity. Similar to NO_x_, SO_2_ may also alter adipocyte differentiation and function, leading to adipocyte hypertrophy and adipose tissue inflammation [52]. Furthermore, SO_2_-induced inflammation may impair insulin signaling pathways in adipose tissue, exacerbating insulin resistance and metabolic dysfunction. Also similar to NO_2_, SO_2_ exposure has been associated with endothelial dysfunction and impaired vascular health and may influence the gut microbiota composition, which can impact energy metabolism and adiposity [46].

A potential reason behind PAHs’ influence on childhood obesity is their ability to disrupt endocrine signaling pathways. PAHs can act as endocrine disruptors, interfering with hormone synthesis, secretion, transport, and receptor binding. These disruptions can lead to the dysregulation of hormones involved in energy balance and metabolism, such as leptin, adiponectin, insulin, and thyroid hormones, which may contribute to adiposity and weight gain in children [59]. Furthermore, PAHs are known carcinogens and can induce oxidative stress and inflammation in various tissues. Oxidative stress and inflammation triggered by PAH exposure can disrupt metabolic homeostasis, leading to insulin resistance, dyslipidemia, and impaired glucose metabolism, all of which contribute to adiposity and weight gain in children [57]. Additionally, PAHs have been associated with alterations in the gut microbiota composition, which can influence the energy harvest from the diet and adiposity. Dysbiosis induced by PAH exposure may promote inflammation and metabolic disturbances, further contributing to obesity development in children [63]. Lastly, PAH exposure has been linked to respiratory conditions such as asthma exacerbations and airway inflammation in children. Respiratory distress caused by PAH exposure may lead to decreased physical activity levels and exercise intolerance, which are risk factors for obesity. PAHs exposure overall leads to altered DNA methylation levels of genes related to inflammation and immune responses, oxidative stress, cell cycle regulation, and signal transduction [62].

### 4.2. The Link between Neonatal Exposure and Childhood Obesity

Maternal exposure to air pollution during pregnancy is a source of various negative health outcomes after birth. While children have certain barriers protecting the respiratory system from absorbing the particles, the fetus relies solely on placental blood exchange, which leads to a higher concentration and longer exposure. The direct placental translocation of chemical particles might impede brain development, raise the risk of autoimmune diseases and allergies, and initiate oxidative stress pathways, resulting in endocrine abnormalities such as diabetes or obesity in children. Studies have shown that mothers living in polluted environments tend to give birth to offspring of an abnormal body mass (significantly low in the first few weeks and rapidly growing after) and heightened blood pressure. Particles absorbed by the placenta and transported by the umbilical blood initiate the creation of reactive oxygen species, which strongly influence the mitochondria and ionic balance across cytoplasmic membranes. The results are visible as delayed nerve impulses, incorrect detoxification, and cell death. Moreover, disruptions of glucose metabolism, decreased heart functions, and insulin resistance directly lead to overweight and obesity in children. At the same time, our review has shown that longitudinal studies on mother–child pairs in the context of exposure to air pollution are scarce.

## 5. Conclusions and Future Management Strategies

From systemic inflammation and the disruption of metabolic homeostasis to alterations in adipose tissue biology and neuroendocrine pathways, air pollution exerts a profound impact on childhood obesity through various pathways.

Addressing environmental and socio-economic determinants of childhood obesity is of crucial importance. Children living in areas with high levels of air pollution face unique challenges in maintaining healthy lifestyles, including limited access to outdoor play spaces, healthy food options, and healthcare resources. Efforts aimed at reducing air pollution levels and promoting healthy lifestyle behaviors are crucial for mitigating the impact of air pollution on childhood obesity [13].

Moving forward, longitudinal studies and interdisciplinary approaches, especially in places with higher levels of air pollution, are needed to address the complex interplay between environmental, social, and individual factors influencing childhood obesity. It is therefore crucial to conduct preclinical experimental studies, as understanding the molecular mechanisms underlying the toxic effects of air pollutants is essential for developing effective preventive and therapeutic strategies. Collaborative efforts between researchers, policymakers, healthcare professionals, and community stakeholders are essential for developing effective strategies for combatting childhood obesity in the context of air pollution [7,44].

Overall, the multifaceted nature of the relationship between air pollution and childhood obesity underscores the importance of adopting a holistic approach to addressing both environmental and socio-economic determinants of health. Based on the reviewed manuscripts, efforts to mitigate air pollution levels and promote healthy lifestyle behaviors are crucial for preventing childhood obesity and improving the long-term health outcomes of children worldwide.

## Figures and Tables

**Table 1:** Significance of PM, SO_2_, NO_2_, and PAHs reflected by their cover environmental and industrial sources, routes of exposure, and adverse health effects [19,20,21,22,23,24].

Air Pollutant	Source	Routes of Exposure	Health Effects
PM_1_	emissions from factories, vehicle exhaust, tire particles from vehicle use, wildfires or indoor wood-burning	ingestion and inhalation	heart attacks, lung cancer, dementia, emphysema, edema
PM_2.5_	motor combustion power plant combustion industrial processes stoves, fireplaces, and home wood burning smoke from fireworks and wildfires, smoking, dust, soot, dirt, windblown salt	ingestion, inhalation, dermal contact	heart and lung disease bronchitis emphysema nonfatal heart attacks irregular heartbeat asthma and more intense flareups decreased lung function early death
PM_10_	smoke, dust and dirt from unsealed road, construction, landfill, and agriculture mold smoke from wildfire and waste burning power generation motor vehicle exhaust	inhalation	lung tissue damage asthma heart failure cancer adverse birth outcomes chronic obstructive pulmonary disease (COPD) premature death
NO_x_	combustion of fossil fuels, road transport	inhalation	premature death, cardiopulmonary effects, decreased lung function growth in children, respiratory symptoms, allergic responses.
SO_2_	fossil fuels combustion, smelting of mineral ores	Inhalation	Bronchitis, cardiovascular disease
PAHs	coal gasification plants, smokehouses, municipal incinerators	ingestion, inhalation, and dermal contact	cataracts, kidney and liver damage, and jaundice, skin inflammation

**Table 3:** Associations between PM_1_ exposure and childhood obesity.

Author, Year	Location	Age [Years]	Cohort Size	Biomarkers	Main Findings
Zhang, 2021 [53]	China	10–18	9897	Glucose oxidase, TG, FBG, HDL-C, Abdominal obesity	Odds ratio of MetS was associated with a 10 μg/m^3^ increase in the two-year average—1.20 higher for abdominal obesity; a 10 µg/m^3^ increase in PM_1_ and NO_2_ was associated with a prevalence of FBG
Zhou, 2021 [51]	China	0–1	10,547	Weight and length, weight for length (WFL), and body mass index (BMI), followed by the definition of overweight and obesity (OWOB)	Positive associations of prenatal PM_1_ and PM_2.5_ exposure with WFL and BMI Z-scores for one-year-old children, which could be partly explained by preterm birth. A 10 μg/m^3^ increase in prenatal exposure to PM_1_ was significantly associated with a 0.105 increase in the WFL Z-score for one-year-old children. Similar associations were found for the BMI Z-score.
Zhang, 2021 [38]	China	7–18	44,718	BMI, waist circumference, WHtR, BMI Z-score	Exposure to PM_1_, associated with an increased BMI Z-score, waist circumference, and WHtR and a higher prevalence of both general and central obesity. Stronger associations were observed for particles, especially PM_1_, than for gaseous pollutants, e.g., NO_2_. Generally, particles with a smaller diameter had greater effect estimates than larger particles.
Wu, 2022 [39]	China	6–18	47,990	Child age- and sex-specific z-scores for body mass index (BMI Z-score) and weight status	Exposure was associated with a greater childhood BMI Z-score and a higher likelihood of obesity. Similar associations were observed for PM_1_, PM_2.5_, and PM_10_ and were greater in boys and children living close to roadways

**Table 4:** Associations between PM_10_ exposure and childhood obesity.

Author, Year	Country	Age [Years]	Cohort Size	Biomarkers	Main Findings
Fioravanti, 2018 [49]	Italy	infants, 4- and 8-year checkup	719	BMI Z-scores, waist circumference, waist-to-hip ratio, and total and HDL cholesterol measured at 8 years.	No association between exposure to vehicular traffic and exposure to pollutants on obesity-related parameters such as BMI, blood lipids, and abdominal adiposity during childhood.
de Bont, 2019 [41]	Spain	7–10	2660	Child weight and height and age- and sex-specific z-scores for body mass index (zBMI)	An IQR increase in PM_10_-home (5.6 μg/m^3^) was associated with a 10% increase in the odds of being overweight or obese.
de Bont, 2021 [46]	Spain	2–5	416,955	Height and weight measures.	A total of 142,590 (34.2%) children developed overweight or obesity. Increased exposure to PM_10_ was associated with a 2–3% increased risk of developing overweight and obesity. For all air pollutants, associations were stronger among children living in the most deprived areas compared to the least deprived areas.
Zhang, 2021 [53]	China	7–18	44,718	Body mass index (BMI), waist circumference, WHtR, and the prevalence of general and central obesity	Exposure to PM_10_ was associated with an increased BMI Z-score, waist circumference, and WHtR and a higher prevalence of both general and central obesity. Particles with a smaller diameter had greater effect estimates than larger particles. The associations were more pronounced in boys than in girls, except for general obesity.

**Table 5:** Associations between NO_x_ + SO_2_ exposure and childhood obesity.

Author, Year	Location	Age [Years]	Cohort Size	Biomarkers	Main Findings
Fioravanti, 2018 [49]	Italy	0 with a follow-up at 4 and 8	500	waist circumference, waist-to-hip ratio, total and HDL cholesterol	No evidence of an association was found between exposure to air pollutants and overweight/obesity in children.
Zhang, 2021 [53]	China	7–18	44,718	BMI Z-score, waist circumference, and WHtR	Exposure to NO_2_ was associated with an increased BMI Z-score, waist circumference, and WHtR and a higher prevalence of both general and central obesity. Generally, stronger associations were observed for particles than for gaseous pollutants, e.g., NO_2_.
Frondelius, 2018 [55]	Sweden	fetal life and 4	5815	body mass index adjusted for Swedish children	No marked associations between traffic-related air pollution (NO_x_) exposure during fetal life and overweight and obesity at age four in Malmö, Sweden, an area with low to medium pollution levels, averaging around 20 µg/m^3^ between 1999 and 2005. Weak evidence of an association between NO_x_ and underweight.
Huang, 2018 [56]	Hong Kong	infants, follow-up at 9, 11, 13, 15	8327	BMI	A higher exposure to NO_2_ in childhood was associated with a higher BMI at ~9, ~13, and ~15 years, a lower exposure to SO_2_ in utero was associated with a lower BMI at ~13 and ~15 years, and a lower exposure to SO_2_ in childhood was associated with a lower BMI at ~15 years. These sex-specific findings concern the relation of air pollution constituents with adiposity.
Zhang, 2021 [38]	China	10–18	9897	glucose oxidase, TG, FBG, HDL-C, abdominal obesity	The odds ratio of Metabolic Syndrome was associated with a 10 μg/m^3^ increase in the two-year average of 1.33; for abdominal obesity, a higher increase in PM_1_ and NO_2_ was associated with the prevalence of FBG.

**Table 6:** Associations between PAHs exposure and childhood obesity.

Author, Year	Location	Age [Years]	Cohort Size	Biomarker	Main Findings
Poursafa, 2018 [57]	Iran	6–18	186		An increased risk of cardiometabolic risk factors and excess weight was associated with exposure to most of the evaluated PAHs. Exposure to 1-hydroxypyrene was associated with a higher risk of cardiometabolic risk factors in participants with excess weight. Exposure to 2-Naphtol was also associated with a higher risk of cardiometabolic risk factors in both groups, but the associations were not significant (*p* < 0.1). For participants without cardiometabolic risk factors, exposure to 2-naphtol, 9-phenanthrol, and ∑ OH-PAH was associated with an increased risk of obesity.
Bushnik, 2020 [58]	Canada	3–18	3667	Urinary PAHs and Waist-to-Height Ratio	Overall, those in the highest quartile for naphthalene or total PAH metabolites had three times greater odds of having central obesity compared with those in the lowest quartile. Urinary PAH metabolites are associated with WHtR, an indicator of central obesity and a predictor of health risks associated with obesity in children as young as 3–5. Urinary PAH metabolites were associated with measures of obesity in children as young as 3–5 years. While there is considerable interest in evaluating the potential role of early-life exposures in disease, PAHs and air pollution more broadly remain understudied risk factors for later-life obesity and its associated comorbidities.
Sears, 2019 [62]	USA	newborns	198 (HOME) and n = 459 (CCAAPS)		Did not find that traffic-related air pollution exposure was associated with a lower birthweight or higher childhood adiposity at an age of 7–8 years in these two cohorts or the pooled sampled. The results should be cautiously interpreted given prior research and the limitations of our study, including the modest sample size, the relatively low ECAT concentrations, and our ability to examine ECAT only during the prenatal period.
Shi, 2022 [59]	USA	5–14	535	BMI, BMI-Z score	Positive association of air PAH contamination with childhood BMI at the age of 5–10 The incidence of obesity was 20.5% at the age of 5 and 33% at age of 11. In addition, the growth trajectory increased across follow-up visits until age 11.
Bushnik, 2023 [63]	Canada	3–18	3667	BMI, WC, and WHtR	BMI, WC, and WHtR were positively associated with total PAH and naphthalene metabolites in the total population aged 3–18 and in the age groups 6–11 and 12–18. In 3–5-year-olds, WHtR, but not BMI, was significantly associated with total PAH, naphthalene, and phenanthrene metabolites. Overall, those in the highest quartile for naphthalene or total PAH metabolites had three-times-greater odds of having central obesity compared with those in the lowest quartile. Urinary PAH metabolites are associated with WHtR, an indicator of central obesity and predictor of health risks associated with obesity, in children as young as 3–5.
Mann, 2021 [60]	USA	6–8	299	HbA1c	Children exposed to higher PAH contamination had an increased level of included HbA1c, systolic blood pressure, and oxidative stress. The results suggested that both short- and longer-term estimated individual-level outdoor residential exposures to several traffic-related air pollutants, including ambient PAHs, are associated with biomarkers of risk for metabolic syndrome and oxidative stress in children.
Li, 2021 [64]	USA	12–19	827	FBG	Among 827 adolescents, 183 (22.13%) had metabolic syndrome. The levels of 2-hydroxynaphthalene (2-NAP), 2-hydroxyphenanthrene (2-PHE), 2-hydroxyfluorene (2-FLU), 1-hydroxynaphthalene (1-NAP), 3-hydroxyfluorene (3-FLU), and 1-hydroxypyrene (1-PYR) were higher in the group of adolescents with metabolic syndrome. There were positive associations between higher concentrations of 2-NAP and 2-FLU and the odds of metabolic syndrome after adjustment. PAHs may be associated with the odds of metabolic syndrome as well as individual metabolic syndrome components among adolescents.
Kim, 2023 [65]	China	6–12	6499	z-BMI	Indoor air pollution exposures were positively associated with higher obese anthropometric indices and increased odds of overweight/obesity in Chinese schoolchildren. Children exposed to ≥three types of indoor air pollutants had a higher z-BMI and a higher risk of overweight/obesity. A dose–response relationship was discovered between the IAP exposure index and z-BMI as well as overweight/obesity.

## Data Availability

Not applicable.
